# Derivation and Validation of a Predictive Model for Advanced Colorectal Neoplasia Among Average‐Risk Adults in China

**DOI:** 10.1002/ijc.70533

**Published:** 2026-05-01

**Authors:** Yan Liu, Yanxiu Liu, Yukun Feng, Boyu Liu, Wenxuan Yan, Nan Zhang, Youhua Lu

**Affiliations:** ^1^ Shandong Cancer Hospital and Institute Shandong First Medical University and Shandong Academy of Medical Sciences Jinan Shandong China; ^2^ School of Public Health Shandong First Medical University and Shandong Academy of Medical Sciences Jinan Shandong China; ^3^ School of Public Health Shandong Second Medical University Weifang Shandong China

**Keywords:** advanced neoplasia, average‐risk population, colorectal cancer screening, predictive model, risk prediction

## Abstract

As the burden of colorectal cancer (CRC) continues to rise and current screening methods have limitations in efficiency and accuracy, there is an urgent need particularly in China and other large populations to develop and validate precision risk prediction models based on individual risk factors with improved discrimination and generalizability to optimize the allocation of screening resources. Asymptomatic individuals aged 40–74 years were recruited from multiple hospitals in China, between January 2024 and May 2025. All participants completed a standardized questionnaire, physical measurements, and colonoscopy. A multivariable logistic regression model was derived to predict the risk of advanced colorectal neoplasia (AN), and a scoring system was derived from regression coefficients. Model performance was evaluated using discrimination, calibration, and risk stratification ability. Among 9617 participants, 673 AN cases were identified. The final model included six variables: age, gender, smoking, drinking, chronic appendicitis, and hypertension. The model demonstrated acceptable discriminatory ability (*c*‐statistics: 0.656 internal validation) and good calibration. A scoring system (range: 0–37) classified individuals into low‐, intermediate‐, and high‐risk groups. AN prevalence for each group was 3.46%, 6.25%, and 11.48%, respectively. The number needed to screen (NNS) improved significantly from 28.9 in the low‐risk group to 8.7 in the high‐risk group. The prediction model and scoring system developed enable effective risk stratification and are suitable for individualized assessment prior to colonoscopy in resource‐limited settings, thereby improving screening efficiency.

## Abstract (highlights)

What's new?

Efficient pre‐colonoscopy risk stratification tools are needed, especially in China. Using multicenter colorectal cancer screening data from Shandong Province, the authors developed and validated a risk prediction model for advanced colorectal neoplasia in asymptomatic individuals using sociodemographic characteristics, lifestyle factors, and medical history. The model achieved acceptable discrimination and effective risk stratification. Advanced neoplasia prevalence ranged from 3.46% to 11.48% across risk groups, reducing the number of individuals who need to be screened to detect one case of advanced neoplasia from 28.9 to 8.7. The model could potentially help optimize screening strategies and guide the allocation of limited colonoscopy resources.

## Graphical abstract

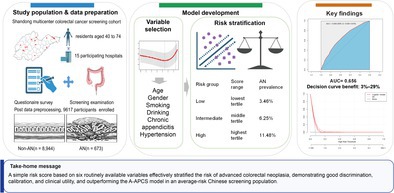

**Abbreviations**
AA: advanced adenoma
A‐APCS: adjusted Asia‐Pacific Colorectal Screening
AN: advanced colorectal neoplasia
BMI: body mass index
CIs: confidence intervals
CRC: colorectal cancer
DCA: decision curve analysis
FIT: fecal immunochemical testing
LASSO: Least Absolute Shrinkage and Selection Operator
NNS: number needed to screen
ORs: odds ratios
RCS: Restricted Cubic Spline
UEBMI: Urban Employee Basic Medical Insurance
URRBMI: Urban–Rural Resident Basic Medical Insurance
VIFs: Variance Inflation Factors

## 1 Introduction

Colorectal cancer (CRC) has become the third most common cancer globally and the second leading cause of cancer‐related mortality [1]. Although CRC screening has been widely proven to reduce morbidity and mortality [2, 3, 4], existing screening methods, such as fecal immunochemical testing (FIT) and colonoscopy, suffer from limitations in applicability and accuracy, resulting in inefficiencies in screening [5, 6]. In particular, some low‐risk populations undergo colonoscopy but yield few positive results, wasting medical resources [7]. Conversely, high‐risk individuals may only receive non‐invasive FIT testing, leading to missed diagnoses of early‐stage lesions and the loss of optimal intervention opportunities [8].

With the advent of the era of precision medicine, CRC prediction models based on individual risk factors have gradually emerged as a potential solution to improve screening efficiency. Over the past decade, several predictive tools have attempted to assess the risk of CRC based on individual risk factors, resulting in *c*‐statistics ranging from 0.64 to 0.80 [9, 10, 11]. Although these models have achieved certain success in Western countries, they often achieve limited risk discrimination ability and poor generalizability. As a populous country, China was experiencing a steady increase in the incidence of CRC each year [12]. Influenced by multiple factors such as dietary habits, lifestyle, and genetic background, the incidence trends of CRC in China were markedly different from those in Western countries [13, 14].

Although some studies have attempted to develop screening tools suitable for the Chinese population, they were largely based on a limited set of risk factors (such as age, gender, and tumor family history) and did not fully consider the combined effects of multidimensional risk factors [15, 16]. While some risk scoring systems have been applied for colorectal cancer screening in the Asia‐Pacific region, most of these systems focused primarily on assessing the risk of colorectal cancer and lacked effective prediction for advanced adenomas (AA) [17]. As direct precursor lesions of CRC, early screening of AA plays a crucial role in reducing the incidence of CRC [18, 19]. Therefore, there is an urgent need to develop a predictive model based on the Chinese population that comprehensively considers advanced neoplasia (AN, including CRC and AA), to improve screening efficiency and enable more precise identification of high‐risk individuals.

To address the inefficiencies and resource waste in the current screening system, this study aimed to identify multidimensional variables closely associated with AN and derive a predictive model that integrates various risk factors for AN. Additionally, a scoring system will be constructed based on individual risk factors, categorizing individuals into high‐, intermediate‐, and low‐risk groups. This will provide crucial support for optimizing screening strategies and the rational allocation of medical resources.

## 2 Methods

### 2.1 Study Population

We conducted this multicenter study across 15 municipal and county hospitals in Shandong Province, China, between January 2024 and May 2025. Asymptomatic, average‐risk individuals aged between 40 and 74 years were enrolled in this study. Participants completed a questionnaire at local hospital screening offices and were excluded if they had a prior history of CRC. All participants also underwent a colonoscopy.

### 2.2 Candidate Predictors and Outcome

Within the Shandong cohort, sociodemographic characteristics, personal and family medical history, and lifestyle habits were obtained through a standardized questionnaire. Anthropometric measurements (height, weight, waist, and hip circumference) were obtained on site by trained staff. Self‐reported values were used only when direct measurements were unavailable. Colonoscopies were performed according to standardized protocols across participating centers.

Demographic and anthropometric variables included gender, age, ethnicity (Han vs. Other), education level (classified as primary school or below, junior high, senior high/vocational, or associate degree or above), marital status (married vs. divorced/widowed/single), household size (1–2, 3–4, or ≥ 5 members), and annual household income (low [< 60,000 CNY], medium [60,000–99,999 CNY], or high [≥ 100,000 CNY]). Occupation was categorized as physical labor, mental labor, or other (including service/self‐employed, unemployed, and retired). Medical insurance was grouped into Urban‐Rural Resident Basic Medical Insurance (URRBMI), Urban Employee Basic Medical Insurance (UEBMI), or other types.

BMI was calculated as weight in kilograms divided by height in meters squared. BMI was analyzed as a continuous variable. Central obesity was defined using sex‐specific waist circumference thresholds: > 90 cm for men and > 85 cm for women, with lower values considered normal.

Lifestyle habits included drinking water source (treated vs. untreated/natural sources), smoking, drinking, physical activity, and sleep habits. Smoking was categorized based on cumulative pack‐years into never, < 30, or ≥ 30 pack‐years, with ≥ 30 pack‐years widely regarded as a marker of heavy smoking exposure and used in clinical guidelines to define high‐risk populations [20]. Drinking was classified based on daily alcohol intake into never, < 15, or ≥ 15 g/day. The ≥ 15 g/day threshold aligns with recommendations in the Chinese Dietary Guidelines (2016) for limiting alcohol intake to reduce health risks [21]. Sensitivity analyses were conducted using alternative smoking and drinking classifications to evaluate the robustness of variable selection with the LASSO procedure.

Physical activity was assessed by the weekly duration of physical labor (continuous hours/week) and exercise frequency (rarely, monthly, or weekly). Sleep behaviors included nightly sleep duration (hours/day), napping habits (no, summer only, other seasons, or year‐round), and nap duration.

Dietary habits were assessed based on the frequency of consumption of specific foods, including fresh vegetables, fresh fruits, meat/dairy products, legumes, pickled foods, fried foods, hot beverages, and moldy foods. Intake frequency was categorized into three levels: never (0 times/week), rarely (< 2 times/week), and often (≥ 2 times/week). Besides, tea consumption (green and black tea) was classified as never, former, or current. Eating behaviors included salt preference (light, medium, and salty), spicy food consumption (yes/no), staple food texture (liquid, soft, and chewy), and meal duration (1–10, 11–20, 21–30, or > 30 min).

Personal medical history included history of *H. pylori* infection, previous FIT, and specific gastrointestinal conditions (ulcerative colitis, Crohn's disease, atypical colitis, hemorrhoids, polyps/adenomas, chronic constipation, chronic diarrhea, mucus bloody stool, chronic appendicitis, and chronic cholecystitis). Comorbidities (specifically hypertension, hyperlipidemia, coronary heart disease, stroke, diabetes, and rheumatic/autoimmune diseases) were recorded and aggregated into a comorbidity score (0, 1, or ≥ 2 conditions). Family history of CRC in first‐degree relatives was also recorded (1, or ≥ 2).

Colonoscopy and pathology reports were reviewed and coded by trained personnel blinded to the questionnaire data. The primary outcome was advanced colorectal neoplasia (AN) [22, 23], defined as the presence of either CRC or AA. CRC included invasive adenocarcinoma, specific subtypes such as neuroendocrine tumors, and non‐epithelial malignancies such as gastrointestinal stromal tumors. AA was identified based on any of the following criteria: (1) diameter ≥ 10 mm; (2) villous or tubulovillous histology; or (3) high‐grade intraepithelial neoplasia (including high‐grade dysplasia and intramucosal adenocarcinoma) [24]. All diagnoses were confirmed through colonoscopy and histopathological examination.

### 2.3 Statistical Analysis

Prior to model development, missing data were handled according to predefined, variable‐specific rules. For continuous variables, variables with a missing rate ≤ 30% were imputed using the mean value, whereas variables with a missing rate > 30% or implausible values were either imputed as zero (if clinically meaningful) or excluded from further analysis. For categorical variables, missing values were imputed using the mode of each variable. It should be noted that mean/mode imputation has inherent limitations, as it may underestimate variability and bias associations. Sensitivity analyses using only complete cases are presented in Table S12 and Figure S10, showing that the predictive performance decreased by only an acceptable 0.004. Participants with logically inconsistent responses (e.g., current smokers without smoking amount or duration, or drinkers without frequency or quantity information) were excluded. The final analysis dataset contained no substantial missing data.

The primary objective of the analysis was to derive a predictive model for identifying individuals with AN based on candidate variables. After data cleaning and variable preprocessing, variable selection was performed using least absolute shrinkage and selection operator (LASSO) logistic regression with 10‐fold cross‐validation to reduce overfitting and address collinearity among candidate predictors. The penalty parameter (*λ*) was selected according to cross‐validated performance and clinical interpretability, and predictors with non‐zero coefficients at the selected *λ* were retained.

Restricted cubic spline (RCS) analysis was used to assess potential non‐linear associations of continuous predictors with AN; based on the spline results (Figure S1), age was modeled using 10‐year categories (≤ 49, 50–59, 60–69, and ≥ 70 years). Multicollinearity was assessed using variance inflation factors (VIFs), and no substantial collinearity was observed among the retained predictors (Table S3). The selected predictors were entered into a multivariable logistic regression model to estimate adjusted odds ratios (ORs) with 95% confidence intervals (CIs).

The predictive performance of the final logistic regression model was evaluated in the full analytic cohort, and model discrimination was quantified using the *c*‐statistic. Calibration was examined using calibration plots, the Hosmer‐Lemeshow goodness‐of‐fit test, and bootstrap internal validation (1000 resamples) to obtain optimism‐corrected performance metrics (including *c*‐statistic, calibration intercept and slope, and Brier score). Clinical utility was assessed using decision curve analysis (DCA). The threshold probability range where the model yielded greater Net Benefit than treat‐all and treat‐none strategies was identified, and the threshold maximizing incremental Net Benefit (ΔNB) was reported.

Based on the multivariable model, we developed a point‐based risk scoring system using the method described by Sullivan et al. [25]. Regression coefficients were standardized by dividing each coefficient by the smallest absolute coefficient in the model, and the resulting values were rounded to integers to derive an additive risk score. Individual total scores were calculated by summing points across predictors and were categorized into low‐, intermediate‐, and high‐risk groups based on the observed risk gradient. The number needed to screen (NNS) was calculated for each group. The TRIPOD checklist for reporting prediction model studies is provided in the Supporting Information. All analyses were performed using R software (version 4.4.2). A two‐sided *p* value < 0.05 was considered statistically significant.

## 3 Results

We enrolled 9658 eligible and consenting participants between January 2024 and May 2025. Participants with incomplete smoking exposure (*n* = 13) or alcohol consumption information (*n* = 28) were excluded, yielding a final analytic cohort of 9617 participants (673 with AN and 8944 without). The baseline demographic and clinical characteristics of participants are presented in Table S1. From 55 candidate variables encompassing demographic characteristics, lifestyle factors, dietary habits, comorbidities, and family history, variable selection was performed using LASSO regression with a prespecified penalty parameter (*λ* = 0.016). Six variables were retained for model development: age, sex, smoking status, alcohol consumption, chronic appendicitis, and hypertension (Table S2).

RCS analysis indicated a linear relationship between age and AN (Figure S1). To facilitate clinical application, age was modeled using 10‐year categories (≤ 49, 50–59, 60–69, and ≥ 70 years), with ≤ 49 years as the reference. Sensitivity analysis treating age as a continuous variable yielded similar effect estimates and model discrimination (Table S11 and Figure S9), supporting the use of categorized age for the final score. In the final multivariable logistic regression model (Table 1), increasing age showed a strong dose–response association with AN risk. Compared with participants aged ≤ 49 years, the odds ratios (ORs) were 2.08 (95% CI: [1.57–2.79]) for ages 50–59, 2.61 (1.97–3.50) for ages 60–69, and 4.50 (3.25–6.28) for ages ≥ 70 (all *p* < 0.001). Male was associated with a higher AN risk (OR = 1.68, 95% CI: [1.39–2.03]; *p* < 0.001). Heavy alcohol consumption (≥ 15 g/day) was also independently associated with AN (OR = 1.58, 95% CI: [1.20–2.05]; *p* < 0.001), whereas light drinking showed no significant association. Although smoking status was not significantly associated with AN after multivariable adjustment, sensitivity analyses demonstrated that smoking was robustly selected by the established LASSO procedure (Tables S13–S18) and had only a minimal impact on predictive performance (Figures S11 and S12). A history of chronic appendicitis was associated with an increased risk of AN (OR = 2.59, 95% CI: [1.35–4.62]; *p* = 0.002). Hypertension showed a modest but statistically significant association (OR = 1.22, 95% CI: [1.01–1.46]; *p* = 0.039). The complete multiple logistic regression equation is as follows:

$$\begin{array}{c} \log\left(\frac{p}{1-p}\right)=-3.7743+0.7318\cdot {\mathrm{Age}}_{50-59}\\ \;+0.9594\cdot {\mathrm{Age}}_{60-69}+1.5040\cdot {\mathrm{Age}}_{\geq 70}\\ \;+0.5179\cdot \text{Male}+0.0856\cdot {\text{Smoking}}_{<30\text{pack}-\text{years}}\\ \;+0.1145\cdot {\text{Smoking}}_{\geq 30\text{pack}-\text{years}}\\ \;-0.0009\cdot {\text{Alcohol}}_{<15\sim g/\mathrm{day}}\\ \;+0.4540\cdot \text{Alcohol}\geq 15\sim g/\mathrm{day}\\ \;+0.9513\cdot \text{Chronic appendicitis}\\ \;+0.1944\cdot \text{Hypertension} \end{array}$$

**TABLE 1:** Variables in the multivariable model (*N* = 6917).

Variables	*N* (%)	OR (95% CI)	*p*
Age group (years)			
≤ 49	1939 (20.16)	—	
50–59	3590 (37.33)	2.079 (1.569–2.794)	< 0.001
60–69	3250 (33.79)	2.61 (1.973–3.504)	< 0.001
≥ 70	838 (8.71)	4.5 (3.253–6.277)	< 0.001
Gender			
Female	5367 (55.81)	—	
Male	4250 (44.19)	1.679 (1.388–2.028)	< 0.001
Smoking			
Never	7795 (81.05)	—	
< 30 pack‐years	1337 (13.90)	1.089 (0.862–1.372)	0.470
≥ 30 pack‐years	485 (5.04)	1.121 (0.807–1.536)	0.485
Drinking			
Never	8792 (91.42)	—	
< 15 g/day	152 (1.58)	0.999 (0.516–1.762)	0.998
≥ 15 g/day	673 (7.00)	1.575 (1.201–2.046)	< 0.001
Chronic appendicitis			
No	9534 (99.14)	—	
Yes	83 (0.86)	2.589 (1.346–4.616)	0.002
Hypertension			
No	7753 (80.62)	—	
Yes	1864 (19.38)	1.215 (1.007–1.458)	0.039

**FIGURE 1:**
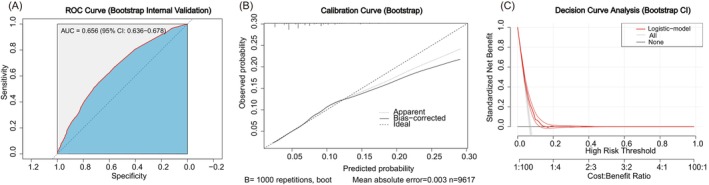
Performance evaluation of the six‐variable discrimination model for advanced colorectal neoplasia. (A) Receiver operating characteristic (ROC) curves. The discrimination (solid line) achieved an *c*‐statistic of 0.656 (95% CI: [0.636–0.678]). (B) Calibration plot. The curve illustrates the agreement between predicted probabilities (*x*‐axis) and observed frequencies (*y*‐axis) of AN. The diagonal gray line represents perfect calibration. Model calibration was further quantified by the Brier score and calibration intercept and slope, with optimism‐corrected estimates obtained using bootstrap resampling. The optimism‐corrected Brier score was 0.064, with a calibration intercept of −0.114 and a calibration slope of 0.954, indicating good overall calibration. The Hosmer‐Lemeshow test (*χ* ^2^ = 2.69, *p* = 0.85) also suggested an adequate goodness‐of‐fit. (C) Decision curve analysis (DCA). The graph depicts the clinical net benefit (*y*‐axis) plotted against threshold probabilities (*x*‐axis). The risk score demonstrated a positive net benefit across a wide range of threshold probabilities (3%–29%) compared with the screen‐all and screen‐none strategies.

The final model demonstrated moderate discrimination, with an apparent *c*‐statistic of 0.656 (95% CI: [0.636–0.678]; Figure 1A). After bootstrap optimism correction, the *c*‐statistic was 0.651, with a Brier score of 0.064. Calibration was satisfactory, as indicated by a Hosmer‐Lemeshow goodness‐of‐fit test (*p* = 0.85) and visual agreement between predicted and observed risks on the calibration plot (Figure 1B). The optimism‐corrected calibration slope was 0.95, indicating minimal overfitting. DCA demonstrated a net clinical benefit of the model across a wide range of threshold probabilities. The model outperformed both the treat‐all and treat‐none strategies for thresholds between 3.0% and 29.0% (Figure 1C). The maximum ΔNB = 0.0159 was observed at a threshold probability of 7.0%, supporting the model's potential utility for risk‐based screening decisions.

Based on the final model, a point‐based scoring system was developed, with a total score ranging from 0 to 37. The point assignments for each predictor are detailed in Table 2. Risk scores were summed across all variables, and participants were stratified into three groups according to score percentiles: low‐risk group (score ≤ 9), intermediate‐risk group (score 10–15), and high‐risk group (score ≥ 16), as shown in Table 3.

**TABLE 2:** Points for each variable.

Variable	Points
Age group (years)	≤ 49: 0; 50–59: 9; 60–69: 11; ≥ 70: 18
Gender	Female: 0; male: 6
Smoking	Never: 0; < 30 pack‐years: 1; ≥ 30 pack‐years: 1
Drinking	Never: 0; < 15 g/day: 0; ≥ 15 g/day: 5
Chronic appendicitis	No: 0; yes: 11
Hypertension	No: 0; yes: 2
Score could range from	0–43 (actual range: 0–37)

**TABLE 3:** Risk of advanced neoplasia by risk group.

Risk group	Score	*n*	AN	Risk AN (%)	NNS
High	≥ 16	3240	372	11.48	8.7
Intermediate	10–15	2881	180	6.25	16
Low	≤ 9	3496	121	3.46	28.9

*Note:* AN indicates advanced neoplasia. Risk AN (%) represents the proportion of individuals with advanced neoplasia in each risk group. NNS (Number Needed to Screen) was calculated as the reciprocal of the AN risk in each group and represents the estimated number of individuals who need to be screened to detect one case of advanced neoplasia.

A clear dose–response relationship was observed between the total risk score and AN prevalence. When grouped into five categories (0–5, 6–10, 11–15, 16–20, and ≥ 21), AN prevalence increased from approximately 2% in the lowest category to over 15% in the highest (Figure S13). Consistently, higher score thresholds were associated with increased specificity and positive predictive value but reduced sensitivity (Table S4), supporting the ability of the scoring system to stratify AN risk. The prevalence of AN increased across the three risk categories. The prevalence was 3.46% in the low‐risk group, 6.25% in the intermediate‐risk group, and 11.48% in the high‐risk group. The distribution of participants across the three groups was 36.35%, 29.96%, and 33.69%. The NNS to detect one case of AN ranged from 28.9 (low‐risk group) to 8.7 (high‐risk group).

To illustrate the practical application of the scoring system, consider a hypothetical 65‐year‐old male participant. He has a history of smoking (< 30 pack‐years), consumes alcohol moderately (< 15 g/day), and has hypertension, but has no history of chronic appendicitis. His total risk score is calculated as follows: 11 points (Age 60–69) + 6 points (Male) + 1 point (Smoking < 30 pack‐years) + 0 points (Drinking < 15 g/day) + 0 points (No chronic appendicitis) + 2 points (Hypertension) = 20 points. According to the Point‐to‐Risk Table (Table S4), a total score of 20 stratifies an individual into the high‐risk group, corresponding to an 11% predicted probability of AN, indicating that colonoscopy screening is recommended for this individual.

These findings demonstrate that the point‐based scoring system can effectively stratify individuals according to their risk of AN. By identifying higher risk individuals with greater detection efficiency, the model may help optimize the allocation of colonoscopy resources and enhance the effectiveness of population‐based colorectal cancer screening programs.

## 4 Discussion

This study derived a risk prediction model for AN in asymptomatic and average‐risk individuals based on multicenter colorectal cancer screening data from Shandong Province, China. The model was built using readily available variables, including sociodemographic characteristics, lifestyle factors, and medical history, and showed acceptable discrimination and good calibration in internal evaluation based on cross‐validated modeling. A simplified scoring system was constructed to stratify the population into low‐, intermediate‐, and high‐risk categories, with the prevalence of AN increasing progressively across risk groups. This model has the potential to serve as a tool for optimizing screening strategies and guiding the allocation of limited colonoscopy resources, thereby improving the overall effectiveness of colorectal cancer screening.

This study identified older age, male, smoking exposure ≥ 30 pack‐years, daily alcohol intake ≥ 15 g/day, chronic appendicitis, and hypertension as risk factors for AN, partly consistent with previous research findings [26, 27, 28]. Age is one of the most definitive risk factors for colorectal neoplasia. With advancing age, the likelihood of accumulating genetic mutations in intestinal epithelial cells increased, which significantly elevated the risk of developing adenomatous lesions and subsequent malignancies [26]. Large‐scale population cohort studies have also demonstrated that the detection rate of AN is substantially higher in individuals aged 50 years and above compared to younger age groups [29, 30]. Men have a higher incidence of CRC and its precursors (such as AA) than women, which may be associated with differences in androgen levels [31], fat distribution [32], inflammatory status [33], and lifestyle behaviors (such as red meat consumption) [27]. Moreover, smoking, an actionable lifestyle factor, has been consistently associated with increased AN risk [34, 35]. Sensitivity analyses using multiple smoking classification thresholds demonstrated that smoking was robustly selected by the established LASSO procedure. The non‐significant associations observed in all subgroups may be attributable to insufficient numbers of smokers in the positive groups, combined with sex imbalance, leading to limited statistical power. Carcinogens in tobacco may act directly on the intestinal mucosa, promoting adenoma formation and malignant transformation through mechanisms involving genetic mutations and chronic inflammation [35]. A meta‐analysis reported that current smokers have approximately 2.1 times the risk of developing AN compared with never‐smokers, which aligns closely with the OR observed in our study.

Importantly, our results showed that higher alcohol intake (≥ 15 g/day) was associated with an increased risk of AN, whereas lower levels of alcohol consumption were not clearly associated with excess risk. This dose‐dependent pattern is biologically plausible and supported by substantial epidemiologic evidence. Ethanol is metabolized to acetaldehyde, a recognized carcinogen that can form DNA adducts, impair DNA repair mechanisms, and induce oxidative stress in colonic epithelial cells. Chronic alcohol exposure has also been shown to disrupt intestinal barrier function, promote mucosal inflammation, alter gut microbiota composition, and interfere with one‐carbon metabolism, all of which may contribute to colorectal carcinogenesis. Large pooled analyses and meta‐analyses of prospective cohort studies have demonstrated a positive association between alcohol consumption and the risk of advanced colorectal adenomas and cancer, particularly at higher intake levels, lending further support to the observed association in our study [36, 37].

We observed an independent association between chronic appendicitis and an increased risk of advanced neoplasia (AN) (OR = 2.59, 95% CI: [1.35–4.62]). However, evidence linking appendiceal disease with colorectal neoplasia remains inconsistent. Some observational studies have reported an increased incidence of colorectal cancer following appendicitis or appendectomy, particularly for proximal tumors [38]. In contrast, more recent large cohort studies and Mendelian randomization analyses have found no clear causal relationship between appendicitis, appendectomy, and long‐term colorectal cancer risk [39]. Taken together, these findings suggest that appendiceal disease may act as a clinical marker of elevated colorectal neoplasia risk rather than a direct causal factor, and the direction of the association—whether related to appendiceal inflammation or appendectomy—remains uncertain. Given the low prevalence of chronic appendicitis in our cohort, this result should be interpreted cautiously. Consistently, excluding this variable resulted in only a minimal change in model discrimination (Δ*c*‐statistic = 0.001, Figure S6), and the corresponding regression estimates are shown in Table S8.

Additionally, hypertension as significant risk factors for AN frequently co‐occur with cancer. Large‐scale observational cohorts have identified hypertension as the most common comorbidity among cancer patients, with a reported prevalence of approximately 38% [40]. This overlap is not merely coincidental. A meta‐analysis of 13 prospective studies confirmed a robust positive association between hypertension and CRC (Relative Risk = 1.11, 95% CI: [1.01–1.21]) [41]. This strong correlation is likely driven by shared risk factors and overlapping mechanisms, including chronic systemic inflammation, increased reactive oxygen species, and oxidative stress, which play pivotal roles in both carcinogenesis and cardiovascular pathology [42]. In the present study, hypertension was associated with a higher risk of AN (OR = 1.215, 95% CI: [1.007–1.458]), consistent with the previous studies. Sensitivity analyses further supported the robustness of this association by demonstrating consistent risk directions across gender subgroups. Detailed subgroup‐specific estimates and model discrimination in men and women are provided in Tables S6 and S7 and Figures S4 and S5.

Notably, our stratified analysis revealed distinct model performance patterns based on hypertensive status. Specifically, in the hypertensive subgroup (vs. the non‐hypertensive subgroup), several key predictors lost statistical significance, which is likely attributable to metabolic and inflammatory dysregulation that may overshadow the marginal contributions of lifestyle factors such as smoking and alcohol consumption in this subgroup. This observation further indicates an elevated background risk of AN among hypertensive individuals. Interestingly, the subgroup models' discriminative ability (*c*‐statistic) both decreased slightly, with only a 0.03 decline, suggesting that our predictive model maintains robustness and clinical applicability even among patients with elevated baseline metabolic risks. Detailed subgroup‐specific regression results and ROC analyses for participants with and without hypertension are shown in Tables S9 and S10 and Figures S7 and S8.

Although the discriminatory ability of our AN prediction model (*c*‐statistic = 0.656) was considered moderate, it demonstrated acceptable robustness and consistency. The favorable calibration and net clinical benefit observed in DCA further support its potential utility as a tool for decision‐making in auxiliary screening strategies. Compared with other models that incorporate molecular or multimodal features, such as FIT, genetic polymorphisms, or protein biomarkers, with *c*‐statistics reaching up to 0.80 [11], our model offers advantages in simplicity and scalability. For instance, the revised adjusted Asia‐Pacific Colorectal Screening (A‐APCS) score achieved a *c*‐statistic of 0.68 in the Chinese population [10]. Furthermore, the *c*‐statistic of A‐APCS in our data was 0.641 (Figure S2), significantly lower than our AN prediction model (*c*‐statistic = 0.656, DeLong test *p* < 0.05). Net reclassification improvement analysis also supported the incremental predictive value of the LASSO‐6 model over the A‐APCS score (Table S5). Additionally, DCA further demonstrated that our AN prediction model yielded a higher net benefit than the A‐APCS score across three distinct threshold probability ranges: 0.03, 0.05–0.16, and 0.20–0.29 (Figure S3). In addition, a model based on the UK Biobank that integrated genome‐wide polygenic risk scores and nongenetic factors reported *c*‐statistics of 0.693 for men and 0.645 for women [9]. The LiFeCRC model, developed from European cohorts and incorporating 16 lifestyle‐related factors, yielded a Harrell's C‐index of 0.709 [43]. In comparison, our model represents a practical balance between feasibility and screening efficiency, offering a viable pathway toward low‐cost, high‐coverage CRC screening in real‐world settings.

A scoring system was developed from the regression model. As the risk score increased, the prevalence of AN rose from 3.46% to 11.48%, accompanied by improved screening efficiency, with NNS decreasing from 28.9 to 8.7. This stratification trend was consistent with findings by Imperiale et al. [44], whose scoring system showed AN risk probabilities of 2.73%, 5.57%, and 25.8% in the low‐, intermediate‐, and high‐risk groups, respectively. Although the variable complexity differed, our scoring system‐based solely on questionnaire‐derived information‐exhibited a comparable trend in risk separation, suggesting high clinical interpretability and credibility. Importantly, this system does not rely on laboratory or genetic indicators, making it particularly suitable for use in primary care settings as a pre‐colonoscopy risk assessment tool. In resource‐limited environments, it offers a feasible solution to support precision screening and efficient resource allocation.

This study has several notable strengths. The data were derived from an average‐risk population in China, with internal validation performed. The model was constructed using simply obtainable and low‐cost variables, making it highly feasible for implementation in primary care and resource‐limited settings. Additionally, the point‐based scoring system enabled a clear gradient in AN risk stratification. Notably, our findings suggested that the predictive model remained robust and clinically applicable, even in patients with elevated baseline metabolic risks. Nonetheless, the study has certain limitations. The model has not yet been externally validated in an entirely independent cohort. Therefore, its robustness and generalizability remain to be confirmed. Moreover, the underlying biological mechanisms for some variables have not yet been conclusively established, and residual confounding cannot be entirely ruled out. Future studies could focus on validating the model in diverse populations and further exploring the biological pathways linking behavioral factors to colorectal neoplasia, while maintaining the model's simplicity and ease of use.

## 5 Conclusion

This study derived and interval validated a predictive model for advanced neoplasia in average‐risk individuals using multicenter data from China and developed a corresponding risk scoring system. The model demonstrated reasonable discrimination, calibration, and stratification performance, making it suitable for implementation in primary care and resource‐limited settings. The scoring system can be used for individualized assessment prior to colonoscopy, thereby improving screening efficiency. Future studies should aim to validate the model in external populations and further investigate the potential mechanisms linking behavioral factors to colorectal neoplasia.

## Author Contributions

**Yan Liu:** conceptualization, methodology, data curation, visualization, writing – original draft, writing – review and editing, conceptualization, methodology, data curation, investigation, writing – original draft, writing – review and editing. **Yanxiu Liu:** conceptualization, methodology, data curation, investigation, writing – original draft, writing – review and editing. **Yukun Feng:** conceptualization, investigation, writing – original draft, writing – review and editing, data curation. **Boyu Liu:** investigation, writing – review and editing. **Wenxuan Yan:** investigation, writing – review and editing. **Nan Zhang:** conceptualization, writing – review and editing, methodology, data curation. **Youhua Lu:** conceptualization, data curation, funding acquisition, writing – review and editing.

## Funding

This work was supported by the National Natural Science Foundation of China (72574131) and the Collaborative Academic Innovation Project of Shandong Cancer Hospital (TS001).

## Ethics Statement

The study protocol was approved by the Medical Ethics Committee of the Cancer Hospital of Shandong First Medical University (Approval No. SDTHEC2024003167) and was conducted in accordance with the principles of the Declaration of Helsinki. Written informed consent was obtained from all participants prior to enrollment. In addition, this study was registered with the Chinese Clinical Trial Registry (ChiCTR2500113249).

## Conflicts of Interest

The authors declare no conflicts of interest.

## Supporting information


**Table S1:** Baseline table.
**Table S2:** Variables selected by LASSO regression.
**Table S3:** Assessment of multicollinearity using variance inflation factors (VIF).
**Table S4:** Distribution of the risk score and diagnostic performance for predicting advanced colorectal neoplasia across different score thresholds.
**Table S5:** Net reclassification improvement (NRI) analysis comparing LASSO‐6 and A‐APCS models.
**Table S6:** Multivariable logistic regression analysis of risk factors for advanced neoplasia in the male subgroup.
**Table S7:** Multivariable logistic regression analysis of risk factors for advanced neoplasia in the female subgroup.
**Table S8:** Multivariable logistic regression analysis of risk factors for advanced neoplasia non‐chronic appendicitis.
**Table S9:** Multivariable logistic regression analysis of risk factors for advanced neoplasia in the hypertension subgroup.
**Table S10:** Multivariable logistic regression analysis of risk factors for advanced neoplasia in the non‐hypertension subgroup.
**Table S11:** Multivariable logistic regression analysis of risk factors for advanced neoplasia utilizing age as a continuous variable.
**Table S12:** Multivariable logistic regression of risk factors for advanced neoplasia (complete case analysis).
**Table S13:** Distribution of study participants by smoking status using 20 pack year cutoff.
**Table S14:** Variables selected by LASSO regression (smoking status using 20 pack‐year cutoff).
**Table S15:** Multivariable logistic regression of risk factors for advanced neoplasia (smoking status using 20 pack‐year cutoff).
**Table S16:** Distribution of study participants by smoking status using 10 pack year cutoff.
**Table S17:** Variables selected by LASSO regression (smoking status using 10 pack‐year cutoff).
**Table S18:** Multivariable logistic regression of risk factors for advanced neoplasia (smoking status using 10 pack‐year cutoff).
**Figure S1:** Restricted cubic spline showing the unadjusted association between age and risk of AN. The solid line represents the unadjusted odds ratio, and the shaded area indicates the 95% confidence interval. The reference value was set at 57 years. *p* value for non‐linearity = 0.092.
**Figure S2:** The receiver operating characteristic (ROC) comparing the LASSO‐6 model and the A‐APCS score. The *c*‐statistic for the LASSO‐6 model was 0.656, which was significantly higher than that of the A‐APCS score (*c*‐statistic = 0.641). The difference in *c* statistics was assessed using DeLong's test, yielding a statistically significant result (*p* = 0.005).
**Figure S3:** Decision curve analysis (DCA) comparing the LASSO‐6 model and the A‐APCS score. The *y*‐axis represents standardized net benefit, and the *x*‐axis represents the threshold probability for defining high‐risk individuals. The “treat‐all” and “treat‐none” strategies are shown as reference lines. Across most clinically relevant threshold ranges, the LASSO‐6 model demonstrates a higher net benefit than the A‐APCS score, indicating superior clinical utility for guiding risk‐based colonoscopy screening decisions.
**Figure S4:** ijc70533‐sup‐0001‐supinfo.pdf. *c*‐statistic of the prediction model in the male subgroup. The model achieved a *c*‐statistic of 0.624 (95% CI: 0.598–0.653).
**Figure S5:** The ROC of the prediction model in the female subgroup. The model achieved a *c*‐statistic of 0.642 (95% CI: 0.609–0.674).
**Figure S6:** The ROC of the prediction model in the non‐chronic appendicitis subgroup. The model achieved a *c*‐statistic of 0.655 (95% CI: 0.634–0.676), suggesting slightly attenuated but acceptable discrimination in non‐chronic appendicitis participants.
**Figure S7:** The ROC of the Prediction model in the hypertension subgroup. The model achieved a *c*‐statistic of 0.632 (95% CI: 0.593–0.674), suggesting slightly attenuated but acceptable discrimination in hypertension participants.
**Figure S8:** The ROC of the prediction model in the non‐hypertension subgroup. The model achieved a *c*‐statistic of 0.652 (95% CI: 0.628–0.676).
**Figure S9:** The ROC of predictive model utilizing age as a continuous variable. The *c*‐statistic illustrates the discriminative ability of the model when age is entered as a continuous variable. The *c*‐statistic was 0.656 (95% CI: 0.635–0.677). This performance is statistically comparable to the primary model where age was stratified into 10‐year intervals (*c*‐statistic = 0.656; 95% CI: 0.636–0.678). Considering the similar predictive performance and the goal of enhancing clinical utility, the categorical form of age (in 10‐year steps) was selected for the final model construction to simplify the scoring system.
**Figure S10:** The ROC of the prediction model in complete cases. The model achieved a *c*‐statistic of 0.652 (95% CI: 0.630–0.674).
**Figure S11:** The ROC of the prediction model (smoking status using 20 pack year cutoff). The model achieved a *c*‐statistic of 0.655 (95% CI: 0.632–0.677).
**Figure S12:** The ROC of the prediction model (smoking status using 10 pack‐year cutoff). The model achieved a *c*‐statistic of 0.656 (95% CI: 0.635–0.677).
**Figure S13:** Observed prevalence of advanced neoplasia (AN) across predefined risk score groups (0–5, 6–10, 11–15, 16–20, and ≥ 21). Points represent the observed prevalence within each group.

## Data Availability

The datasets generated during and/or analyzed during the current study are available from the corresponding author on reasonable request.
