# Supplementary material for: Derivation and Validation of a Predictive Model for Advanced Colorectal Neoplasia Among Average‐Risk Adults in China
Source: Int J Cancer. 2026 May 1;159(6):1455–63. doi: 10.1002/ijc.70533 (PMC13397183; doi:10.1002/ijc.70533)
Supplement: Supplementary file 1 — Table S1: Baseline table. Table S2: Variables selected by LASSO regression. Table S3: Assessment of multicollinearity using variance inflation factors (VIF). Table S4: Distribution of the risk score and diagnostic performance for predicting advanced colorectal neoplasia across different score thresholds. Table S5: Net reclassification improvement (NRI) analysis comparing LASSO‐6 and A‐APCS models. Table S6: Multivariable logistic regression analysis of risk factors for advanced neoplasia in the male subgroup. Table S7: Multivariable logistic regression analysis of risk factors for advanced neoplasia in the female subgroup. Table S8: Multivariable logistic regression analysis of risk factors for advanced neoplasia non‐chronic appendicitis. Table S9: Multivariable logistic regression analysis of risk factors for advanced neoplasia in the hypertension subgroup. Table S10: Multivariable logistic regression analysis of risk factors for advanced neoplasia in the non‐hypertension subgroup. Table S11: Multivariable logistic regression analysis of risk factors for advanced neoplasia utilizing age as a continuous variable. Table S12: Multivariable logistic regression of risk factors for advanced neoplasia (complete case analysis). Table S13: Distribution of study participants by smoking status using 20 pack year cutoff. Table S14: Variables selected by LASSO regression (smoking status using 20 pack‐year cutoff). Table S15: Multivariable logistic regression of risk factors for advanced neoplasia (smoking status using 20 pack‐year cutoff). Table S16: Distribution of study participants by smoking status using 10 pack year cutoff. Table S17: Variables selected by LASSO regression (smoking status using 10 pack‐year cutoff). Table S18: Multivariable logistic regression of risk factors for advanced neoplasia (smoking status using 10 pack‐year cutoff). Figure S1: Restricted cubic spline showing the unadjusted association between age and risk of AN. The solid line represents the unadj [file IJC-159-1455-s001.pdf]

Derivation and Validation of a Predictive Model for Advanced Colorectal Neoplasia among Average-Risk Adults in China

Yan Liu, Yanxiu Liu, Yukun Feng, Boyu Liu, Wenxuan Yan, Nan Zhang, Youhua Lu

Supplementary Information

Supplementary Table 1. Baseline table
Supplementary Table 2. Variables selected by LASSO regression
Supplementary Table 3. Assessment of multicollinearity using variance inflation factors (VIF)
Supplementary Table 4. Distribution of the risk score and diagnostic performance for predicting advanced colorectal neoplasia across different score thresholds.
Supplementary Table 5. Net Reclassification Improvement (NRI) Analysis Comparing LASSO-6 and A-APCS Models
Supplementary Table 6. Multivariable Logistic Regression Analysis of Risk Factors for Advanced Neoplasia in the Male Subgroup
Supplementary Table 7. Multivariable Logistic Regression Analysis of Risk Factors for Advanced Neoplasia in the Female Subgroup
Supplementary Table 8. Multivariable Logistic Regression Analysis of Risk Factors for Advanced Neoplasia non-Chronic Appendicitis
Supplementary Table 9. Multivariable Logistic Regression Analysis of Risk Factors for Advanced Neoplasia in the Hypertension Subgroup
Supplementary Table 10. Multivariable Logistic Regression Analysis of Risk Factors for Advanced Neoplasia in the non-Hypertension Subgroup
Supplementary Table 11. Multivariable Logistic Regression Analysis of Risk Factors for Advanced Neoplasia utilizing age as a continuous variable
Supplementary Table 12. Multivariable Logistic Regression of Risk Factors for Advanced Neoplasia (Complete Case Analysis)
Supplementary Table 13. Distribution of Study Participants by Smoking Status Using 20 Pack-Year Cutoff
Supplementary Table 14. Variables selected by LASSO regression (Smoking Status Using 20 Pack-Year Cutoff)
Supplementary Table 15. Multivariable Logistic Regression of Risk Factors for Advanced Neoplasia (Smoking Status Using 20 Pack-Year Cutoff)
Supplementary Table 16 Distribution of Study Participants by Smoking Status Using 10 Pack-Year Cutoff
Supplementary Table 17 Variables selected by LASSO regression (Smoking Status Using 10 Pack-Year Cutoff)
Supplementary Table 18. Multivariable Logistic Regression of Risk Factors for Advanced Neoplasia (Smoking Status Using 10 Pack-Year Cutoff)

Supplementary Figure 1. Restricted cubic spline showing the unadjusted association between age and risk of AN.
Supplementary Figure 2. The Receiver Operating Characteristic (ROC) Comparing the LASSO-6 Model and the A-APCS Score.
Supplementary Figure 3. Decision Curve Analysis (DCA) Comparing the LASSO-6 Model and the A-APCS Score..
Supplementary Figure 4. c-statistic of the Prediction Model in the Male Subgroup.
Supplementary Figure 5. The ROC of the Prediction Model in the Female Subgroup.
Supplementary Figure 6. The ROC of the Prediction Model in the non-Chronic Appendicitis Subgroup.
Supplementary Figure 7. The ROC of the Prediction Model in the Hypertension Subgroup.

Supplementary Figure 8. The ROC of the Prediction Model in the non-Hypertension Subgroup.
Supplementary Figure 9. The ROC of predictive model utilizing age as a continuous variable.
Supplementary Figure 10. The ROC of the Prediction Model in complete cases.
Supplementary Figure 11. The ROC of the Prediction Model (Smoking Status Using 20 Pack-Year Cutoff).
Supplementary Figure 12 The ROC of the Prediction Model (Smoking Status Using 10 Pack-Year Cutoff).
Supplementary Figure 13 Observed prevalence of advanced neoplasia (AN) across predefined risk score groups (0-5, 6-10, 11-15, 16-20, and ≥21).
TRIPOD-Checklist 1: Prediction Model Development and Validation

Supplementary Table 1. Baseline table

Characteristic	Overall<br>N = 9,617	control<br>N = 8,944	case<br>N = 673	p-value[1]
**Gender**				<0.001
female	5,367 (56%)	5,093 (57%)	274 (41%)	
male	4,250 (44%)	3,851 (43%)	399 (59%)	
**Age**	57.00 (51.00,64.00)	57.00 (51.00,63.00)	61.00 (54.00,67.00)	<0.001
**Ethnicity**				0.380
Han	9,575 (100%)	8,903 (100%)	672 (100%)	
Other	42 (0.4%)	41 (0.5%)	1 (0.1%)	
**Education**				0.024
Primary/Below	1,909 (20%)	1,786 (20%)	123 (18%)	
Junior High	3,673 (38%)	3,404 (38%)	269 (40%)	
Senior High/Vocational	2,071 (22%)	1,904 (21%)	167 (25%)	
Associate/Above	1,964 (20%)	1,850 (21%)	114 (17%)	
**Marriage status**				0.640
Divorced/Widowed/Single	279 (3%)	257 (3%)	22 (3%)	
Married	9,338 (97%)	8,687 (97%)	651 (97%)	
**occupation**				0.008
Physical labor	4,639 (48%)	4,296 (48%)	343 (51%)	
Mental labor	1,376 (14%)	1,307 (15%)	69 (10%)	
Other	3,602 (37%)	3,341 (37%)	261 (39%)	
**Insurance coverage**				0.780
URRBMI	5,707 (59%)	5,316 (59%)	391 (58%)	
UEBMI	3,822 (40%)	3,546 (40%)	276 (41%)	
Other	88 (1%)	82 (1%)	6 (1%)	
**Household size**				<0.001
1-2	4,515 (47%)	4,150 (46%)	365 (54%)	
3-4	3,922 (41%)	3,686 (41%)	236 (35%)	
5+	1,180 (12%)	1,108 (12%)	72 (11%)	
**Annual household income**				0.470

Characteristic	Overall<br>N = 9,617	control<br>N = 8,944	case<br>N = 673	p-value[1]
Low (<60k)	5,443 (57%)	5,047 (56%)	396 (59%)	
Medium (60-100k)	2,326 (24%)	2,173 (24%)	153 (23%)	
High (≥100k)	1,848 (19%)	1,724 (19%)	124 (18%)	
**BMI**	24.97 (23.15,27.06)	24.97 (23.14,27.06)	25.06 (23.44,27.34)	0.250
**Central obesity**				0.780
Normal	3,503 (36%)	3,254 (36%)	249 (37%)	
High	6,114 (64%)	5,690 (64%)	424 (63%)	
**Smoking**				<0.001
<30 pack-years	1,337 (14%)	1,204 (13%)	133 (20%)	
≥30 pack-years	485 (5%)	430 (5%)	55 (8%)	
Never	7,795 (81%)	7,310 (82%)	485 (72%)	
**Drinking**				<0.001
<15 g per day	152 (2%)	140 (2%)	12 (2%)	
≥15 g per day	673 (7%)	589 (7%)	84 (12%)	
Never	8,792 (91%)	8,215 (92%)	577 (86%)	
**Black tea intake**				0.160
Never	6,541 (68%)	6,097 (68%)	444 (66%)	
Former	70 (0.7%)	68 (0.8%)	2 (0.3%)	
Current	3,006 (31%)	2,779 (31%)	227 (34%)	
**Green tea intake**				0.270
Never	5,773 (60%)	5,378 (60%)	395 (59%)	
Former	49 (0.5%)	48 (0.5%)	1 (0.1%)	
Current	3,795 (39%)	3,518 (39%)	277 (41%)	
**Water source intake**				0.930
Treated	9,247 (96%)	8,599 (96%)	648 (96%)	
Untreated	370 (4%)	345 (4%)	25 (4%)	
**Fresh vegetable intake**				0.075
Never (0×/wk)	14 (0.1%)	14 (0.2%)	0 (0%)	
Rarely (<2×/wk)	88 (1%)	77 (1%)	11 (2%)	

Characteristic	Overall<br>N = 9,617	control<br>N = 8,944	case<br>N = 673	p-value[1]
Often (≥2×/wk)	9,515 (99%)	8,853 (99%)	662 (98%)	
**Fresh fruit intake**				0.430
Never (0×/wk)	43 (0.4%)	38 (0.4%)	5 (0.7%)	
Rarely (<2×/wk)	949 (10%)	879 (10%)	70 (10%)	
Often (≥2×/wk)	8,625 (90%)	8,027 (90%)	598 (89%)	
**Meat/dairy intake**				0.810
Never (0×/wk)	26 (0.3%)	25 (0.3%)	1 (0.1%)	
Rarely (<2×/wk)	525 (6%)	489 (6%)	36 (5%)	
Often (≥2×/wk)	9,066 (94%)	8,430 (94%)	636 (95%)	
**Legume intake**				0.270
Never (0×/wk)	81 (0.8%)	79 (0.9%)	2 (0.3%)	
Rarely (<2×/wk)	2,602 (27%)	2,421 (27%)	181 (27%)	
Often (≥2×/wk)	6,934 (72%)	6,444 (72%)	490 (73%)	
**Pickled food intake**				0.980
Never (0×/wk)	924 (10%)	859 (10%)	65 (10%)	
Rarely (<2×/wk)	6,761 (70%)	6,290 (70%)	471 (70%)	
Often (≥2×/wk)	1,932 (20%)	1,795 (20%)	137 (20%)	
**Fried food intake**				0.180
Never (0×/wk)	604 (6%)	557 (6%)	47 (7%)	
Rarely (<2×/wk)	7,869 (82%)	7,336 (82%)	533 (79%)	
Often (≥2×/wk)	1,144 (12%)	1,051 (12%)	93 (14%)	
**Hot beverage intake**				0.023
Never (0×/wk)	3,702 (38%)	3,421 (38%)	281 (42%)	
Rarely (<2×/wk)	4,880 (51%)	4,572 (51%)	308 (46%)	
Often (≥2×/wk)	1,035 (11%)	951 (11%)	84 (12%)	
**Moldy food intake**				0.290
Never (0×/wk)	9,193 (96%)	8,545 (96%)	648 (96%)	
Rarely (<2×/wk)	388 (4%)	367 (4%)	21 (3%)	
Often (≥2×/wk)	36 (0.4%)	32 (0.4%)	4 (0.6%)	
**staple food texture**				0.210

Characteristic	Overall N = 9,617	control N = 8,944	case N = 673	p-value[1]
Liquid	327 (3%)	309 (4%)	18 (3%)	
Soft	5,612 (58%)	5,233 (59%)	379 (56%)	
Chewy	3,678 (38%)	3,402 (38%)	276 (41%)	
**Eating duration**				<0.001
1to10Min	809 (8%)	721 (8%)	88 (13%)	
11to20Min	5,437 (57%)	5,058 (57%)	379 (56%)	
21to30Min	2,847 (30%)	2,675 (30%)	172 (26%)	
>30Min	524 (5%)	490 (5%)	34 (5%)	
**Food saltiness**				0.019
Medium	5,064 (53%)	4,712 (53%)	352 (52%)	
Light	1,752 (18%)	1,652 (18%)	100 (15%)	
Salty	2,801 (29%)	2,580 (29%)	221 (33%)	
**Eat spicy**	2,368 (25%)	2,207 (25%)	161 (24%)	0.700
**Weekly physical labor(hours)**	10.00 (0.00,30.00)	10.00 (0.00,30.00)	7.00 (0.00,26.00)	0.003
**Physical exercise frequency**				0.480
Rarely	5,575 (58%)	5,190 (58%)	385 (57%)	
Monthly	805 (8%)	755 (8%)	50 (7%)	
Weekly	3,237 (34%)	2,999 (34%)	238 (35%)	
**Sleep duration (night time)**	7.00 (7.00,8.00)	7.00 (7.00,8.00)	7.00 (7.00,8.00)	0.400
**Nap status**				0.045
No	3,781 (39%)	3,532 (39%)	249 (37%)	
Yes (summer only)	1,537 (16%)	1,410 (16%)	127 (19%)	
Yes (other seasons)	1,300 (14%)	1,224 (14%)	76 (11%)	
Yes (year-round)	2,999 (31%)	2,778 (31%)	221 (33%)	
**Nap duration**	0.50 (0.00,1.00)	0.50 (0.00,1.00)	0.50 (0.00,1.00)	0.056
**Hp infection**	309 (3%)	284 (3%)	25 (4%)	0.510
**Stool frequency**				0.210
Once per day	6,715 (70%)	6,246 (70%)	469 (70%)	

Characteristic	Overall<br>N = 9,617	control<br>N = 8,944	case<br>N = 673	p-value[1]
More than once per day	1,790 (19%)	1,662 (19%)	128 (19%)	
Every other day	724 (8%)	683 (8%)	41 (6%)	
Less than 3 times per week	388 (4%)	353 (4%)	35 (5%)	
**Ulcerative colitis**	40 (0.4%)	39 (0.4%)	1 (0.1%)	0.420
**Crohn disease**	1 (<0.1%)	1 (<0.1%)	0 (0%)	>0.99
**Atypical colitis**	23 (0.2%)	22 (0.2%)	1 (0.1%)	0.930
**Hemorrhoids**	1,315 (14%)	1,234 (14%)	81 (12%)	0.220
**Intestinal polyps/adenoma**	580 (6%)	552 (6%)	28 (4%)	0.042
**Chronic constipation**	744 (8%)	697 (8%)	47 (7%)	0.490
**Chronic diarrhea**	590 (6%)	545 (6%)	45 (7%)	0.590
**Mucus bloody stool**	60 (0.6%)	51 (0.6%)	9 (1.3%)	0.029
**Chronic appendicitis**	83 (1%)	70 (1%)	13 (2%)	0.004
**Chronic cholecystitis**	135 (1.4%)	121 (1.4%)	14 (2.1%)	0.170
**FIT history**	348 (4%)	322 (4%)	26 (4%)	0.810
**Hypertension**	1,864 (19%)	1,688 (19%)	176 (26%)	<0.001
**Hyperlipidemia**	822 (9%)	755 (8%)	67 (10%)	0.200
**Coronary heart disease**	263 (3%)	248 (3%)	15 (2%)	0.480
**Stroke**	106 (1%)	95 (1%)	11 (2%)	0.240
**Diabetes**	698 (7%)	635 (7%)	63 (%)	0.035
**Rheumatic autoimmune disease**	68 (0.7%)	62 (0.7%)	6 (0.9%)	0.720
**Comorbidity**				<0.001
0	6,816 (71%)	6,387 (71%)	429 (64%)	
1	2,049 (21%)	1,871 (21%)	178 (26%)	
≥2	752 (8%)	686 (8%)	66 (10%)	
**FDR with CRC**				0.540
0	9,211 (96%)	8,571 (96%)	640 (95%)	
1	402 (4%)	369 (4%)	33 (5%)	
2	4 (<0.1%)	4 (<0.1%)	0 (0%)	

[1]Pearson's Chi-squared test; Welch Two Sample t-test

[2]Data are presented as median (interquartile range) for continuous variables and number (percentage) for categorical variables.
[3]BMI: Body Mass Index; CRC: Colorectal Cancer; FDR: First Degree Relative

Supplementary Table 2. Variables selected by LASSO regression

Variable	Coefficient
Gender male	0.310
Age (per year)	0.0295
Smoking status: Never	−0.0250
Drinking status: ≥15 g/day	0.208
Chronic appendicitis (Yes)	0.0278
Hypertension (Yes)	0.0050

Supplementary Table 3. Assessment of multicollinearity using variance inflation factors (VIF)

Variable	GVIF	Df	GVIF^(1/(2*Df))
Age group	1.046497	3	1.007603
Gender	1.395137	1	1.181159
Smoking	1.420841	2	1.091783
Drinking	1.185717	2	1.043507
Chronic appendicitis	1.005982	1	1.002986
Hypertension	1.034244	1	1.016978

GVIF: generalized variance inflation factor;
Df: degrees of freedom for multi-level categorical variables.
GVIF^(1/(2×Df)) represents the standardized VIF and was used to assess multicollinearity.

Supplementary Table 4. Distribution of the risk score and diagnostic performance for predicting advanced colorectal neoplasia across different score thresholds.

Score	Participants	Score distribution	Cumulative distribution	AN prevalence	Sensitivity	Specificity	PPV (%)	NPV (%)	Youden index
0	1043	10.85	10.85	2.01	100	0	7		0
1	4	0.04	10.89	0	96.88	11.43	7.6	97.99	0.083
2	45	0.47	11.35	2.22	96.88	11.47	7.61	97.99	0.083
5	3	0.03	11.39	0	96.73	11.96	7.64	97.99	0.087
6	402	4.18	15.57	5.22	96.73	12	7.64	97.99	0.087
7	203	2.11	17.68	3.94	93.61	16.26	7.76	97.13	0.099
8	40	0.42	18.09	0	92.42	18.44	7.86	97	0.109
9	1756	18.26	36.35	3.99	92.42	18.88	7.9	97.07	0.113
10	8	0.08	36.44	12.5	82.02	37.73	9.02	96.54	0.197
11	1651	17.17	53.6	5.88	81.87	37.81	9.01	96.52	0.197
12	77	0.8	54.4	3.9	67.46	55.19	10.17	95.75	0.226
13	467	4.86	59.26	7.49	67.01	56.02	10.29	95.76	0.23
14	17	0.18	59.44	0	61.81	60.85	10.62	95.49	0.227
15	661	6.87	66.31	6.66	61.81	61.04	10.66	95.5	0.228
16	375	3.9	70.21	7.47	55.27	67.93	11.48	95.28	0.232
17	730	7.59	77.8	9.73	51.11	71.81	12.01	95.13	0.229
18	750	7.8	85.6	11.47	40.56	79.18	12.79	94.65	0.197
19	172	1.79	87.39	9.88	27.79	86.61	13.5	94.1	0.144
20	354	3.68	91.07	11.02	25.26	88.34	14.01	94.01	0.136
21	114	1.19	92.25	16.67	19.47	91.86	15.25	93.81	0.113

22	88	0.92	93.17	17.05	16.64	92.92	15.03	93.68	0.096
23	154	1.6	94.77	12.99	14.41	93.74	14.76	93.57	0.081
24	177	1.84	96.61	13.56	11.44	95.24	15.31	93.46	0.067
25	154	1.6	98.21	15.58	7.88	96.95	16.26	93.33	0.048
26	63	0.66	98.87	14.29	4.31	98.4	16.86	93.18	0.027
27	52	0.54	99.41	13.46	2.97	99	18.35	93.13	0.02
28	1	0.01	99.42	100	1.93	99.51	22.81	93.1	0.014
29	16	0.17	99.58	18.75	1.78	99.51	21.43	93.09	0.013
30	15	0.16	99.74	13.33	1.34	99.65	22.5	93.07	0.01
31	12	0.12	99.86	33.33	1.04	99.8	28	93.06	0.008
32	8	0.08	99.95	12.5	0.45	99.89	23.08	93.02	0.003
33	1	0.01	99.96	100	0.3	99.97	40	93.02	0.003
35	1	0.01	99.97	0	0.15	99.97	25	93.01	0.001
36	2	0.02	99.99	50	0.15	99.98	33.33	93.01	0.001
37	1	0.01	100	0	0	99.99	0	93	0

Supplementary Table 5. Net Reclassification Improvement (NRI) Analysis Comparing LASSO-6 and A-APCS Models

**Old model**	**New model**	**N**	**Case**	**Bootstrap frequency**	**NRI method**	**NRI**
A-APCS	LASSO-6	9617	673	1000	continuous/category-less (up-down='diff', cut=0)	0.116

Net reclassification improvement (NRI) was calculated to quantify the incremental predictive value of the LASSO-6 model over the A-APCS score. A continuous, category-free NRI approach was applied using the up-down reclassification method, with 1,000 bootstrap resamples to estimate the stability of the NRI.

Supplementary Table 6. Multivariable Logistic Regression Analysis of Risk Factors for Advanced Neoplasia in the Male Subgroup

Variables	OR (95% CI)	P value
Age group (years)		
50-59	2.249 (1.571-3.296)	<0.001
60-69	2.324 (1.617-3.417)	<0.001
≥70	3.810 (2.489-5.904)	<0.001
Smoking status		
<30 pack-years	1.073 (0.848-1.354)	0.553
≥30 pack-years	1.081 (0.773-1.488)	0.641
Drinking status		
<15 g per day	1.026 (0.527-1.823)	0.934
≥15 g per day	1.533 (1.165-1.999)	0.002
Chronic appendicitis (Yes)	3.433 (1.409-7.570)	0.004
Hypertension (Yes)	1.240 (0.976-1.566)	0.074

Supplementary Table 7. Multivariable Logistic Regression Analysis of Risk Factors for Advanced Neoplasia in the Female Subgroup

Variables	OR (95% CI)	P value
Age group (years)		
50-59	1.861 (1.190-3.016)	0.009
60-69	3.080 (1.998-4.944)	<0.001

≥70	5.741 (3.488-9.694)	<0.001
Smoking status		
<30 pack-years	2.026 (0.265-8.862)	0.415
≥30 pack-years	NE	-
Drinking status		
<15 g per day	NE	-
≥15 g per day	1.478 (0.187-7.252)	0.666
Chronic appendicitis (Yes)	1.902 (0.650-4.452)	0.181
Hypertension (Yes)	1.132 (0.834-1.517)	0.416

NE: Not estimable due to sparse data.

Supplementary Table 8. Multivariable Logistic Regression Analysis of Risk Factors for Advanced Neoplasia non-Chronic Appendicitis

Variables	OR (95% CI)	P value
Age group (years)		
50-59	2.193 (1.644-2.971)	<0.001
60-69	2.714 (2.036-3.674)	<0.001
≥70	4.604 (3.304-6.476)	<0.001
Gender (Male)	1.680 (1.388-2.032)	<0.001
Smoking status		
<30 pack-years	1.065 (0.840-1.345)	0.598
≥30 pack-years	1.120 (0.803-1.537)	0.494
Drinking status		
<15 g per day	0.883 (0.429-1.620)	0.710
≥15 g per day	1.565 (1.190-2.038)	0.001
Hypertension (Yes)	1.241 (1.028-1.492)	0.023

Supplementary Table 9. Multivariable Logistic Regression Analysis of Risk Factors for Advanced Neoplasia in the Hypertension Subgroup

Variables	OR (95% CI)	P value
Age group (years)		
50-59	9.033 (2.766-55.607)	0.002
60-69	8.698 (2.671-53.493)	0.003
≥70	12.709 (3.751-79.462)	<0.001
Gender (Male)	1.713 (1.157-2.528)	0.007
Smoking status		
<30 pack-years	0.957 (0.606-1.496)	0.850
≥30 pack-years	1.468 (0.818-2.554)	0.185
Drinking status		
<15 g per day	0.980 (0.283-2.592)	0.970
≥15 g per day	1.519 (0.923-2.442)	0.091
Chronic appendicitis (Yes)	1.340 (0.310-4.044)	0.644

Supplementary Table 10. Multivariable Logistic Regression Analysis of Risk Factors for Advanced Neoplasia in the non-Hypertension Subgroup

Variables	OR (95% CI)	P value
Age group (years)		
50-59	1.788 (1.330-2.437)	<0.001
60-69	2.452 (1.829-3.334)	<0.001
≥70	4.554 (3.199-6.514)	<0.001

Gender (Male)	1.672 (1.344-2.076)	<0.001
Smoking status		
<30 pack-years	1.135 (0.863-1.486)	0.361
≥30 pack-years	0.990 (0.657-1.453)	0.959
Drinking status		
<15 g per day	1.067 (0.470-2.099)	0.863
≥15 g per day	1.580 (1.138-2.162)	0.005
Chronic appendicitis (Yes)	3.545 (1.659-6.875)	<0.001

Supplementary Table 11. Multivariable Logistic Regression Analysis of Risk Factors for Advanced Neoplasia utilizing age as a continuous variable

Variables	OR (95% CI)	P value
Age (continuous)	1.048 (1.038-1.059)	<0.001
Gender (Male)	1.679 (1.388-2.028)	<0.001
Smoking status		
<30 pack-years	1.086 (0.860-1.368)	0.485
≥30 pack-years	1.118 (0.805-1.531)	0.495
Drinking status		
<15 g per day	0.992 (0.513-1.749)	0.979
≥15 g per day	1.569 (1.197-2.038)	<0.001
Chronic appendicitis (Yes)	2.505 (1.303-4.463)	0.003
Hypertension (Yes)	1.203 (0.997-1.445)	0.051

Supplementary Table 12. Multivariable Logistic Regression of Risk Factors for Advanced Neoplasia (Complete Case Analysis)

Variables	OR (95% CI)	P value
Age group (years)		
50-59	2.163 (1.581-3.015)	<0.001
60-69	2.746 (2.012-3.82)	<0.001
≥70	4.478 (3.122-6.495)	<0.001
Gender (Male)	1.564 (1.268-1.926)	<0.001
Smoking status		
<30 pack-years	1.044 (0.806-1.347)	0.741
≥30 pack-years	1.074 (0.751-1.512)	0.687
Drinking status		
<15 g per day	1.165 (0.582-2.111)	0.639
≥15 g per day	1.758 (1.313-2.331)	<0.001
Chronic appendicitis (Yes)	2.297 (1.13-4.257)	0.013
Hypertension (Yes)	1.222 (0.999-1.487)	0.049

Supplementary Table 13. Distribution of Study Participants by Smoking Status Using 20 Pack-Year Cutoff

Smoking status	Overall N = 9,617	Control N = 8,944	Case N = 673	p-value[1]
Never	7,795 (81%)	7,310 (82%)	485 (72%)	
<20 pack-years	1,028 (11%)	935 (10%)	93 (14%)	<0.001
≥20 pack-years	794 (8.3%)	699 (7.8%)	95 (14%)	

[1]Pearson's Chi-squared test; Welch Two Sample t-test

Supplementary Table 14. Variables selected by LASSO regression (Smoking Status Using 20 Pack-Year Cutoff)

Variable	Coefficient
Gender male	0.309
Age (per year)	0.0294
Smoking status: ≥20 pack/year	0.0650
Drinking status: ≥15 g/day	0.205
Chronic appendicitis (Yes)	0.0275
Hypertension (Yes)	0.00581

Supplementary Table 15. Multivariable Logistic Regression of Risk Factors for Advanced Neoplasia (Smoking Status Using 20 Pack-Year Cutoff)

Variables	OR (95% CI)	P value
Age group (years)		
50-59	2.067 (1.56-2.777)	<0.001
60-69	2.58 (1.951-3.463)	<0.001
≥70	4.467 (3.229-6.23)	<0.001
Gender (Male)	1.678 (1.387-2.027)	<0.001
Smoking status		
<20 pack-years	0.987 (0.759-1.276)	0.924
≥20 pack-years	1.239 (0.949-1.607)	0.111
Drinking status		
<15 g per day	1.009 (0.521-1.78)	0.976
≥15 g per day	1.565 (1.194-2.033)	<0.001
Chronic appendicitis (Yes)	2.583 (1.342-4.606)	0.002
Hypertension (Yes)	1.22 (1.011-1.465)	0.035

Supplementary Table 16 Distribution of Study Participants by Smoking Status Using 10 Pack-Year Cutoff

Smoking status	Overall N = 9,617	Control N = 8,944	Case N = 673	p-value[1]
Never	7,795 (81%)	7,310 (82%)	485 (72%)	
<10 pack-years	682 (7.1%)	625 (7.0%)	57 (8.5%)	<0.001
≥10 pack-years	1,140 (12%)	1,009 (11%)	131 (19%)	

Supplementary Table 17 Variables selected by LASSO regression (Smoking Status Using 10 Pack-Year Cutoff)

Variable	Coefficient
Gender male	0.293
Age (per year)	0.0295
Smoking status: ≥10 pack/year	0.120
Drinking status: ≥15 g/day	0.190
Chronic appendicitis (Yes)	0.0218
Hypertension (Yes)	0.00594

Supplementary Table 18. Multivariable Logistic Regression of Risk Factors for Advanced Neoplasia (Smoking Status Using 10 Pack-Year Cutoff)

Variables	OR (95% CI)	P value
Age group (years)		
50-59	2.063 (1.557-2.772)	<0.001
60-69	2.593 (1.962-3.479)	<0.001
≥70	4.527 (3.273-6.313)	<0.001
Gender (Male)	1.679 (1.389-2.029)	<0.001

Smoking status		
<10 pack-years	0.867 (0.631-1.174)	0.367
≥10 pack-years	1.246 (0.982-1.576)	0.068
Drinking status		
<15 g per day	1.003 (0.518-1.771)	0.992
≥15 g per day	1.555 (1.186-2.021)	0.001
Chronic appendicitis (Yes)	2.54 (1.319-4.533)	0.003
Hypertension (Yes)	1.225 (1.016-1.471)	0.031

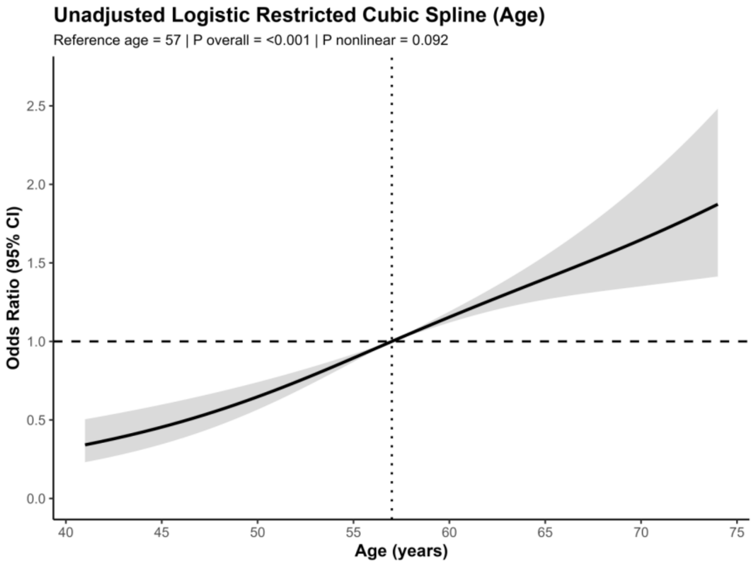

Supplementary Figure 1. Restricted cubic spline showing the unadjusted association between age and risk of AN. The solid line represents the unadjusted odds ratio, and the shaded area indicates the 95% confidence interval. The reference value was set at 57 years. p value for non-linearity = 0.092.

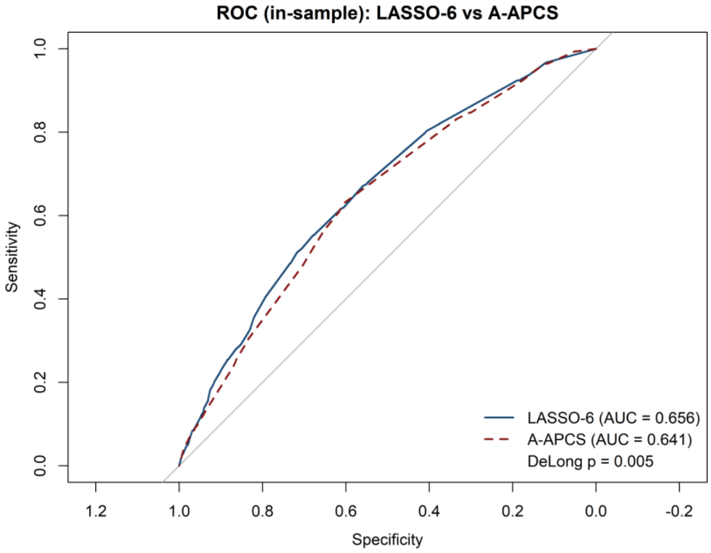

Supplementary Figure 2. The Receiver Operating Characteristic (ROC) Comparing the LASSO-6 Model and the A-APCS Score. The c-statistic for the LASSO-6 model was 0.656, which was significantly higher than that of the A-APCS score (c-statistic = 0.641). The difference in c-statistics was assessed using DeLong’s test, yielding a statistically significant result (p = 0.005).

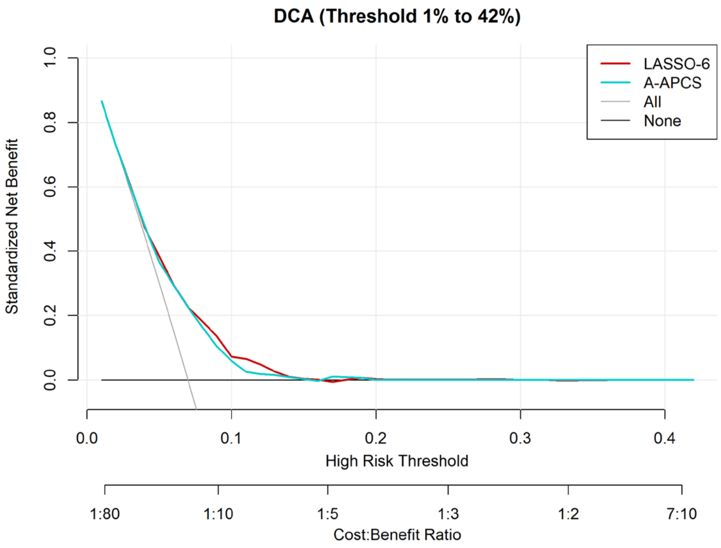

Supplementary Figure 3. Decision Curve Analysis (DCA) Comparing the LASSO-6 Model and the A-APCS Score. The y-axis represents standardized net benefit, and the x-axis represents the threshold probability for defining high-risk individuals. The "treat-all" and "treat-none" strategies are shown as reference lines. Across most clinically relevant threshold ranges, the LASSO-6 model demonstrates a higher net benefit than the A-APCS score, indicating superior clinical utility for guiding risk-based colonoscopy screening decisions.

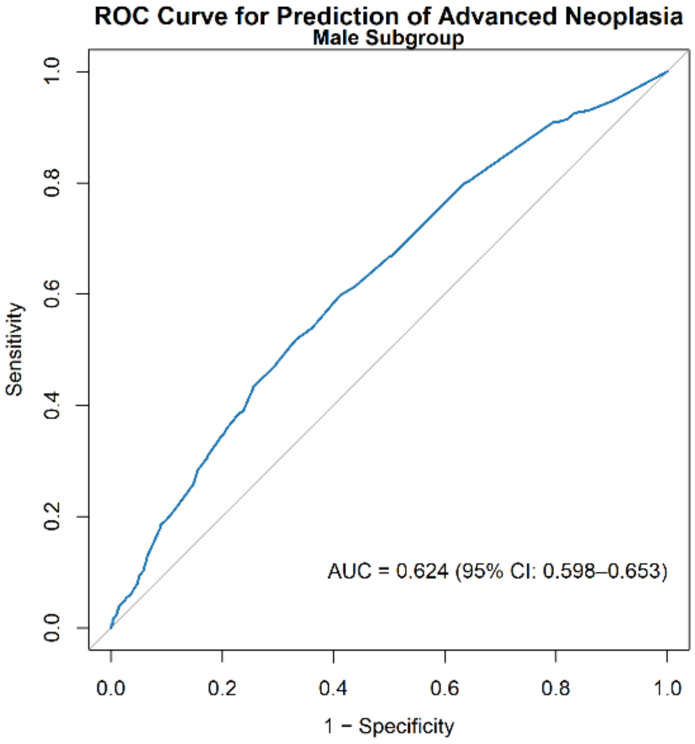

Supplementary Figure 4. c-statistic of the Prediction Model in the Male Subgroup. The model achieved an c-statistic of 0.624 (95% CI: 0.598-0.653).

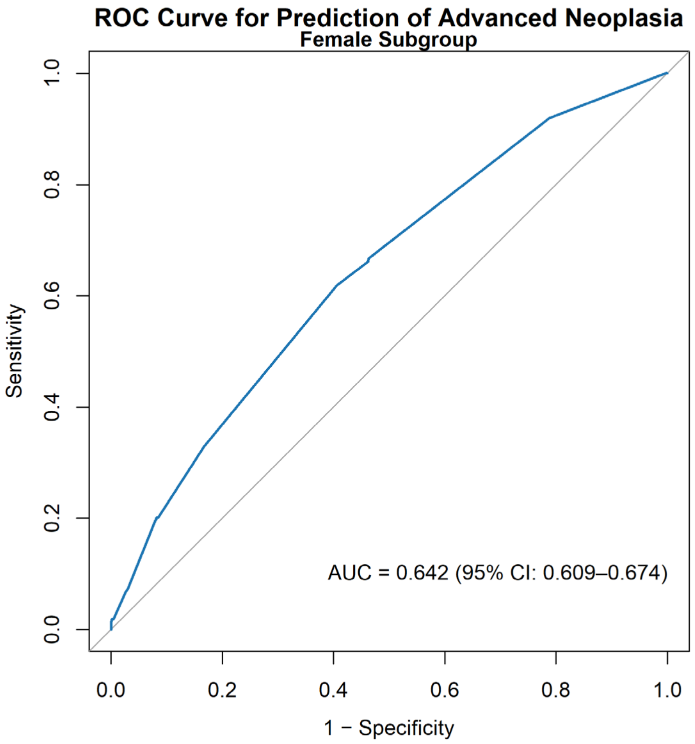

Supplementary Figure 5. The ROC of the Prediction Model in the Female Subgroup. The model achieved an c-statistic of 0.642 (95% CI: 0.609-0.674).

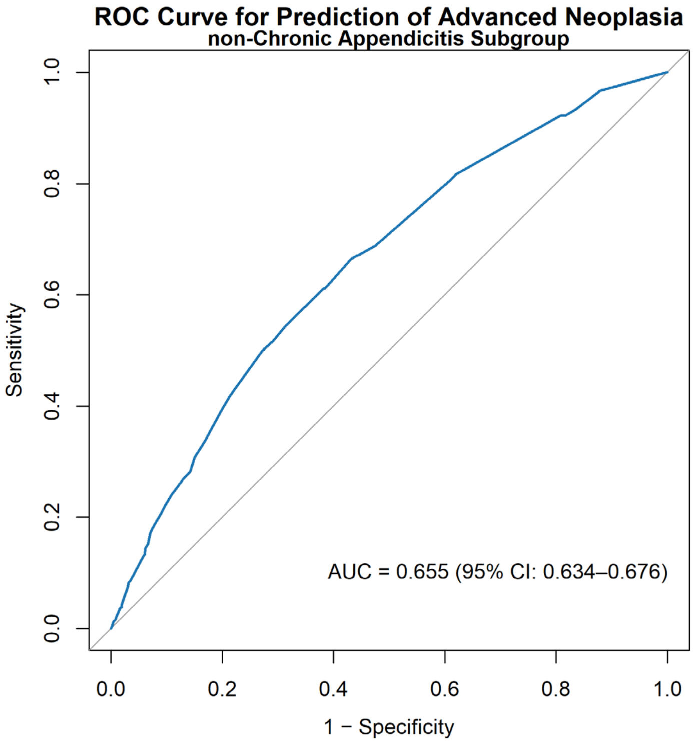

Supplementary Figure 6. The ROC of the Prediction Model in the non-Chronic Appendicitis Subgroup. The model achieved an c-statistic of 0.655 (95% CI: 0.634-0.676), suggesting slightly attenuated but acceptable discrimination in non-chronic appendicitis participants.

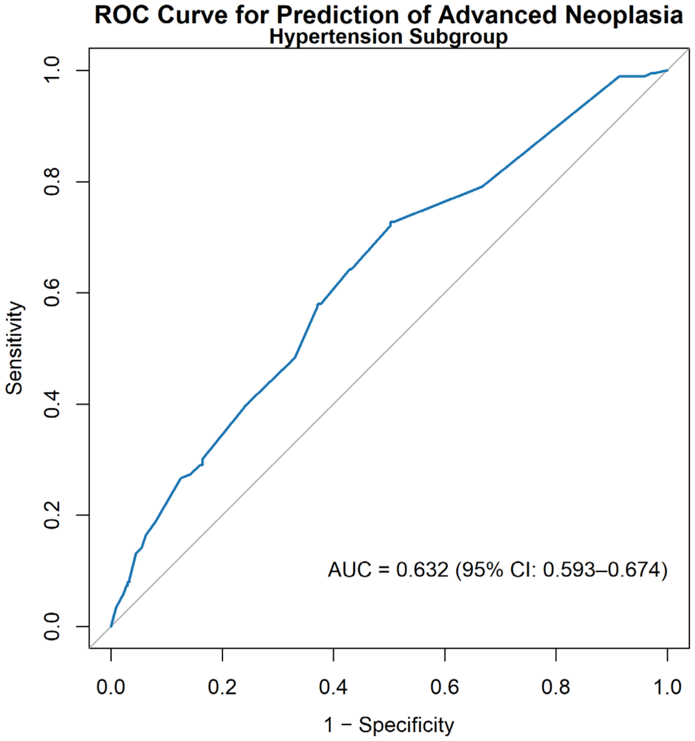

Supplementary Figure 7. The ROC of the Prediction Model in the Hypertension Subgroup. The model achieved an c-statistic of 0.632 (95% CI: 0.593-0.674), suggesting slightly attenuated but acceptable discrimination in hypertension participants.

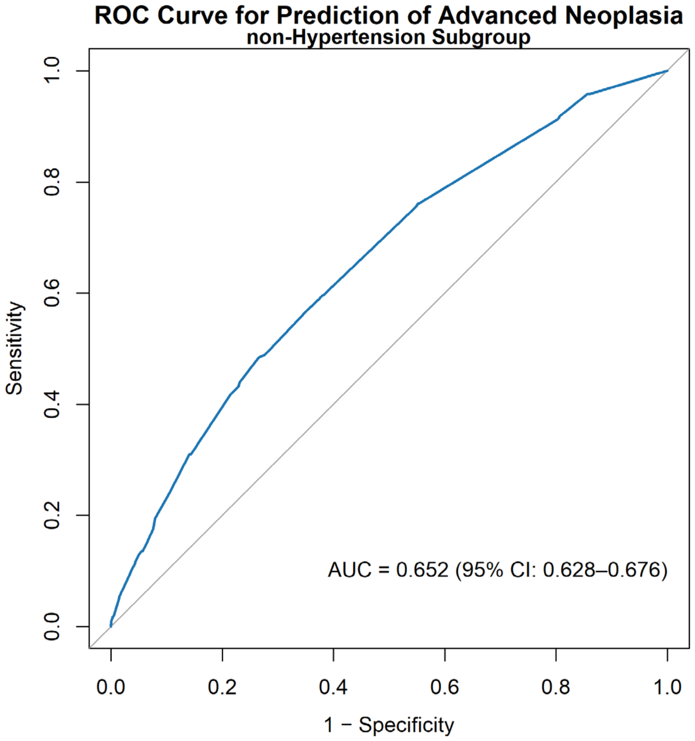

Supplementary Figure 8. The ROC of the Prediction Model in the non-Hypertension Subgroup. The model achieved an c-statistic of 0.652 (95% CI: 0.628-0.676).

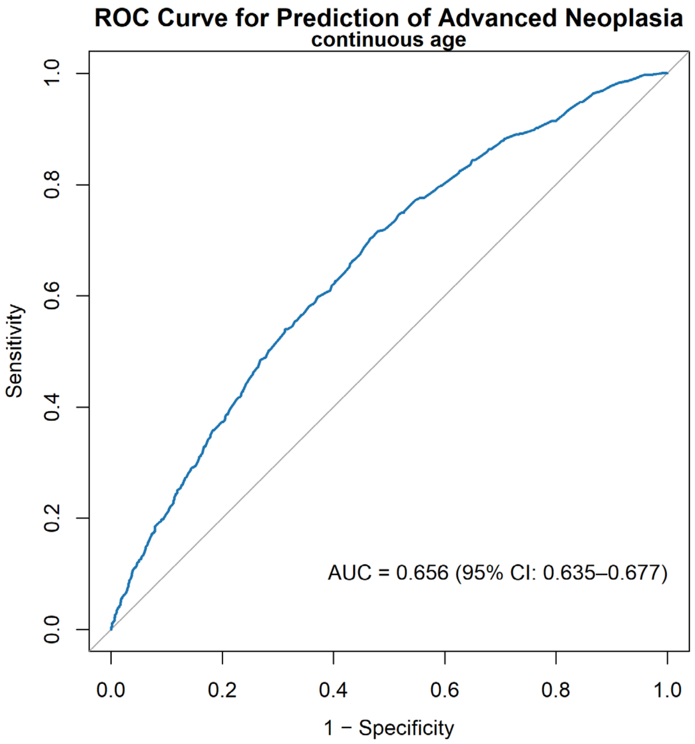

Supplementary Figure 9. The ROC of predictive model utilizing age as a continuous variable. The c-statistic illustrates the discriminative ability of the model when age is entered as a continuous variable. The c-statistic was 0.656 (95% CI: 0.635-0.677). This performance is statistically comparable to the primary model where age was stratified into 10-year intervals (c-statistic = 0.656; 95% CI: 0.636-0.678). Considering the similar predictive performance and the goal of enhancing clinical utility, the categorical form of age (in 10-year steps) was selected for the final model construction to simplify the scoring system.

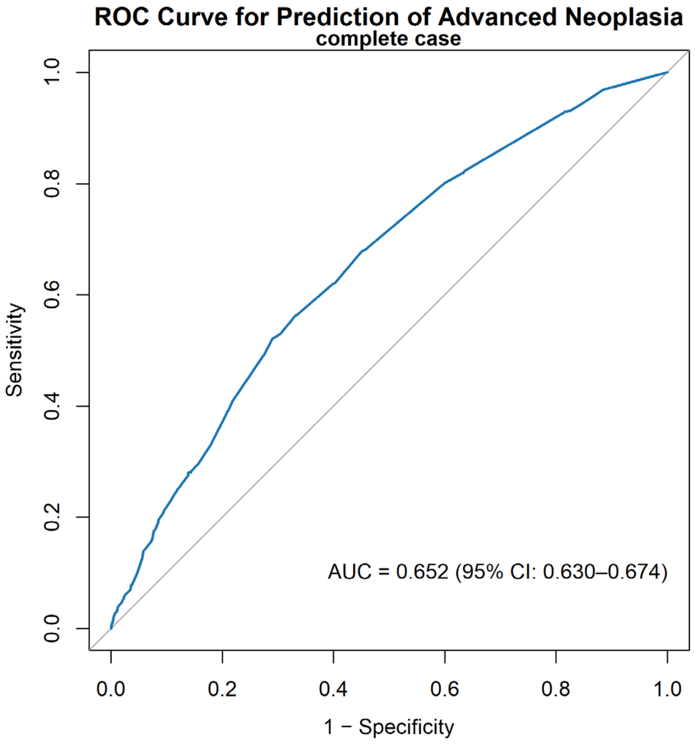

Supplementary Figure 10. The ROC of the Prediction Model in complete cases. The model achieved an c-statistic of 0.652 (95% CI: 0.630-0.674).

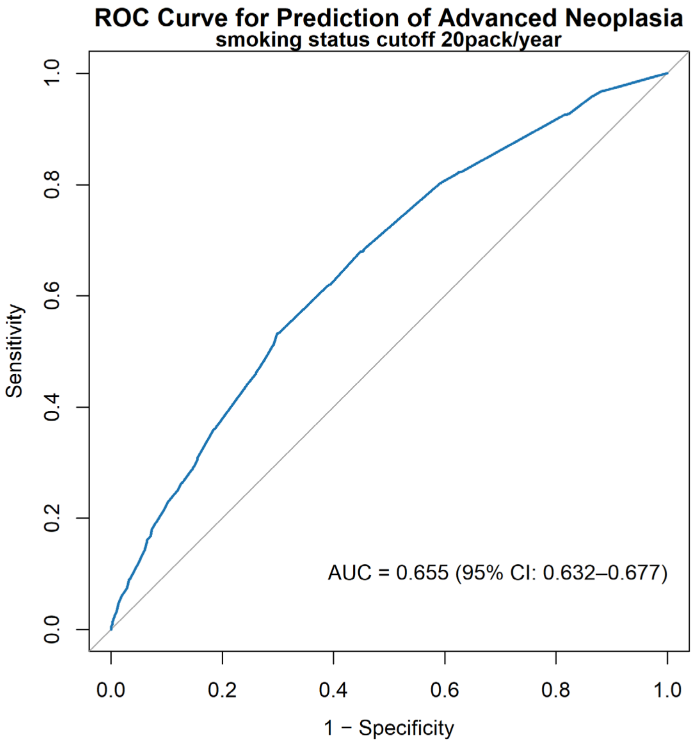

Supplementary Figure 11. The ROC of the Prediction Model (Smoking Status Using 20 Pack-Year Cutoff). The model achieved an c-statistic of 0.655 (95% CI: 0.632-0.677).

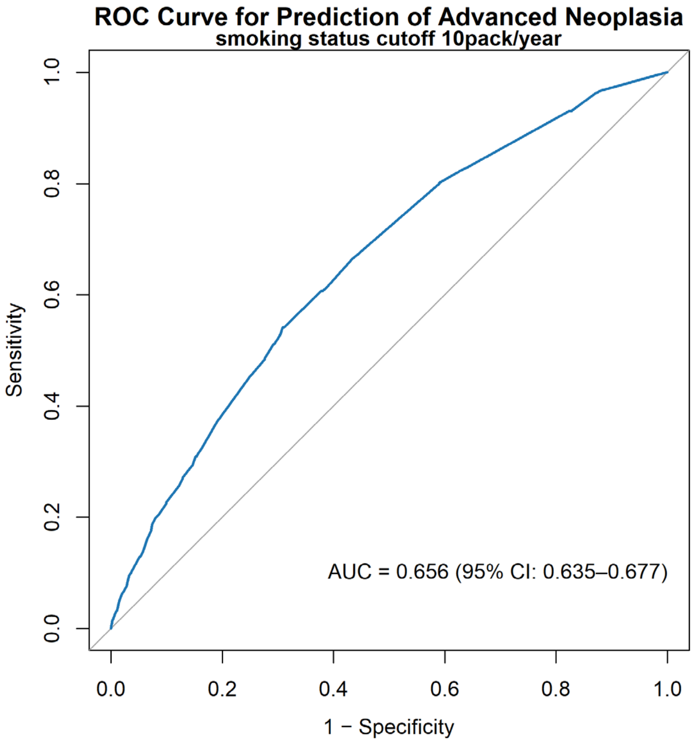

Supplementary Figure 12 The ROC of the Prediction Model (Smoking Status Using 10 Pack-Year Cutoff). The model achieved an c-statistic of 0.656 (95% CI: 0.635-0.677).

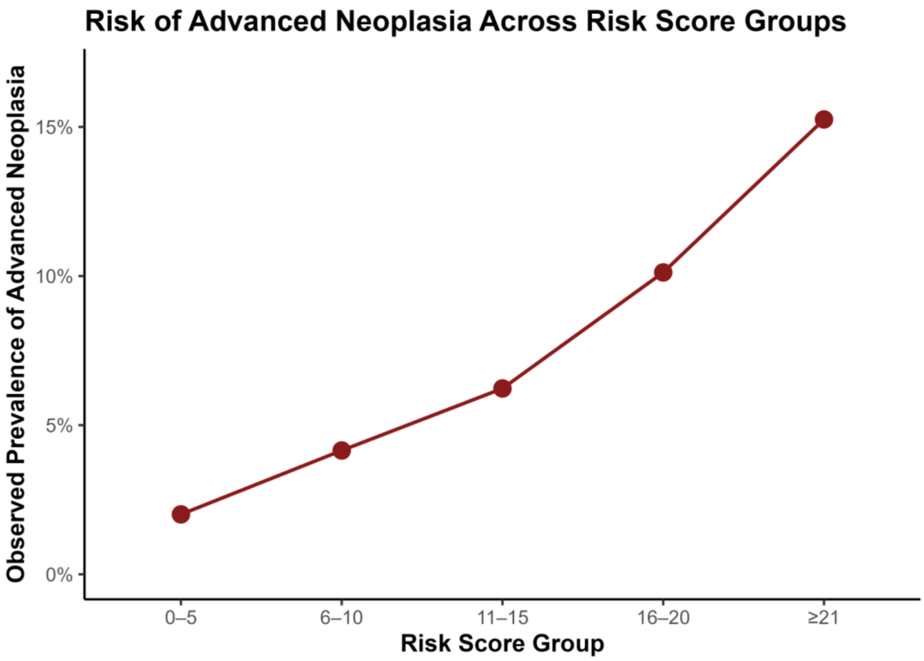

Supplementary Figure 13 Observed prevalence of advanced neoplasia (AN) across predefined risk score groups (0-5, 6-10, 11-15, 16-20, and ≥21). Points represent the observed prevalence within each group.

TRIPOD-Checklist 1: Prediction Model Development and Validation

Section/Topic	Item		Checklist Item	Page
**Title and abstract**				
Title	1	D;V	Identify the study as developing and/or validating a multivariable prediction model, the target population, and the outcome to be predicted.	Page1
Abstract	2	D;V	Provide a summary of objectives, study design, setting, participants, sample size, predictors, outcome, statistical analysis, results, and conclusions.	Page 3
**Introduction**				
Background and objectives	3a	D;V	Explain the medical context (including whether diagnostic or prognostic) and rationale for developing or validating the multivariable prediction model, including references to existing models.	Page 5
	3b	D;V	Specify the objectives, including whether the study describes the development or validation of the model or both.	Page 5
**Methods**				
Source of data	4a	D;V	Describe the study design or source of data (e.g., randomized trial, cohort, or registry data), separately for the development and validation data sets, if applicable.	Page 6
	4b	D;V	Specify the key study dates, including start of accrual; end of accrual; and, if applicable, end of follow-up.	Page 6
Participants	5a	D;V	Specify key elements of the study setting (e.g., primary care, secondary care, general population) including number and location of centres.	Page 6
	5b	D;V	Describe eligibility criteria for participants.	Page 6
	5c	D;V	Give details of treatments received, if relevant.	-
Outcome	6a	D;V	Clearly define the outcome that is predicted by the prediction model, including how and when assessed.	Page 9
	6b	D;V	Report any actions to blind assessment of the outcome to be predicted.	Page 9
Predictors	7a	D;V	Clearly define all predictors used in developing or validating the multivariable prediction model, including how and when they were measured.	Page6-7, page 10
	7b	D;V	Report any actions to blind assessment of predictors for the outcome and other predictors.	Page 9
Sample size	8	D;V	Explain how the study size was arrived at.	-
Missing data	9	D;V	Describe how missing data were handled (e.g., complete-case analysis, single imputation, multiple imputation) with details of any imputation method.	Page 9
Statistical analysis methods	10a	D	Describe how predictors were handled in the analyses.	Page 10
	10b	D	Specify type of model, all model-building procedures (including any predictor selection), and method for internal validation.	Page 10-11
	10c	V	For validation, describe how the predictions were calculated.	Page 10
	10d	D;V	Specify all measures used to assess model performance and, if relevant, to compare multiple models.	Page10, page 17-18
	10e	V	Describe any model updating (e.g., recalibration) arising from the validation, if done.	-
Risk groups	11	D;V	Provide details on how risk groups were created, if done.	Page 11
Development vs. validation	12	V	For validation, identify any differences from the development data in setting, eligibility criteria, outcome, and predictors.	-
**Results**				
Participants	13a	D;V	Describe the flow of participants through the study, including the number of participants with and without the outcome and, if applicable, a summary of the follow-up time. A diagram may be helpful.	Graphical abstract figure
	13b	D;V	Describe the characteristics of the participants (basic demographics, clinical features, available predictors), including the number of participants with missing data for predictors and outcome.	Supplementary table 1
	13c	V	For validation, show a comparison with the development data of the distribution of important variables (demographics, predictors and outcome).	Supplementary table 1
Model development	14a	D	Specify the number of participants and outcome events in each analysis.	Supplementary table 1
	14b	D	If done, report the unadjusted association between each candidate predictor and outcome.	-
Model specification	15a	D	Present the full prediction model to allow predictions for individuals (i.e., all regression coefficients, and model intercept or baseline survival at a given time point).	Page 12
	15b	D	Explain how to the use the prediction model.	Page 14
Model performance	16	D;V	Report performance measures (with CIs) for the prediction model.	Page 13, Figure 2A
Model-updating	17	V	If done, report the results from any model updating (i.e., model specification, model performance).	Figure 2B-2C
**Discussion**				
Limitations	18	D;V	Discuss any limitations of the study (such as nonrepresentative sample, few events per predictor, missing data).	Page 19
Interpretation	19a	V	For validation, discuss the results with reference to performance in the development data, and any other validation data.	-
	19b	D;V	Give an overall interpretation of the results, considering objectives, limitations, results from similar studies, and other relevant evidence.	Page 14-19
Implications	20	D;V	Discuss the potential clinical use of the model and implications for future research.	Page 19-20

Other information				
Supplementary information	21	D;V	Provide information about the availability of supplementary resources, such as study protocol, Web calculator, and data sets.	Supplementary tables and figures
Funding	22	D;V	Give the source of funding and the role of the funders for the present study.	Page21

*Items relevant only to the development of a prediction model are denoted by D, items relating solely to a validation of a prediction model are denoted by V, and items relating to both are denoted D;V. We recommend using the TRIPOD Checklist in conjunction with the TRIPOD Explanation and Elaboration document.
